# Nonlinear spectral mixture effects for photosynthetic/non-photosynthetic vegetation cover estimates of typical desert vegetation in western China

**DOI:** 10.1371/journal.pone.0189292

**Published:** 2017-12-14

**Authors:** Cuicui Ji, Yonghong Jia, Zhihai Gao, Huaidong Wei, Xiaosong Li

**Affiliations:** 1 School of Remote Sensing and Information Engineering, Wuhan University, Wuhan, China; 2 Key Laboratory of Digital Earth Science, Institute of Remote Sensing and Digital Earth, Chinese Academy of Sciences, Beijing, China; 3 Institute of Forest Resources Information Technique, Chinese Academy of Forestry, Beijing, China; 4 State Key Laboratory of Desertification and Aeolian Sand Disaster Combating, Gansu Desert Control Research Institute, Lanzhou, Gansu; IFRS, BRAZIL

## Abstract

Desert vegetation plays significant roles in securing the ecological integrity of oasis ecosystems in western China. Timely monitoring of photosynthetic/non-photosynthetic desert vegetation cover is necessary to guide management practices on land desertification and research into the mechanisms driving vegetation recession. In this study, nonlinear spectral mixture effects for photosynthetic/non-photosynthetic vegetation cover estimates are investigated through comparing the performance of linear and nonlinear spectral mixture models with different endmembers applied to field spectral measurements of two types of typical desert vegetation, namely, *Nitraria* shrubs and *Haloxylon*. The main results were as follows. (1) The correct selection of endmembers is important for improving the accuracy of vegetation cover estimates, and in particular, shadow endmembers cannot be neglected. (2) For both the *Nitraria* shrubs and *Haloxylon*, the Kernel-based Nonlinear Spectral Mixture Model (KNSMM) with nonlinear parameters was the best unmixing model. In consideration of the computational complexity and accuracy requirements, the Linear Spectral Mixture Model (LSMM) could be adopted for *Nitraria* shrubs plots, but this will result in significant errors for the *Haloxylon* plots since the nonlinear spectral mixture effects were more obvious for this vegetation type. (3) The vegetation canopy structure (planophile or erectophile) determines the strength of the nonlinear spectral mixture effects. Therefore, no matter for Nitraria shrubs or Haloxylon, the non-linear spectral mixing effects between the photosynthetic / non-photosynthetic vegetation and the bare soil do exist, and its strength is dependent on the three-dimensional structure of the vegetation canopy. The choice of linear or nonlinear spectral mixture models is up to the consideration of computational complexity and the accuracy requirement.

## Introduction

Arid and semiarid regions occupy 41% of the world's whole land area, where the ecosystem are prone to desertification due to the irrational use of natural resources and climate change[1]. In western China, oases are the basis of human life and social economic development, supporting more than 95% of the population, although they cover less than 5% of the total area of arid regions[2]. Desert vegetation between oasis and desert is very important, which functions as a shelter against drifting sand, and also a major food source for sheep, goats and camels[3]. However, the desert vegetation is threatened by increasing overuse due to rapid population growth, and by an increasing use of water for irrigation. In order to preserve the desert vegetation as a valuable resource and to maintain its sand-fixing and food-providing function, it has to be protected[4]. A sustainable management of desert vegetation requires accurate and timely information on vegetation cover at large scale[5].

From a functional perspective, vegetation can be categorized as photosynthetic (green leaves) and non-photosynthetic (wood, senescent material, and litter) material [6, 7]. Undoubtedly, photosynthetic vegetation (PV) is a critical component of desert vegetation, but it is not the only component. Non-photosynthetic vegetation(NPV) also plays a key role in carbon and nutrient uptake, fire risk and frequency, and wind and water erosion, the potential for fire risk and wind and water erosion[7]. Thus, acquiring the fractional cover of PV (*f*_pv_) and NPV (*f*_npv_) information simultaneously will be of great value for desert vegetation studies. Remote sensing has considerable potential for providing accurate estimates of *f*_pv_ and *f*_npv_ of desert vegetation. Spectral Mixture Analysis (SMA) is a widely used technique to retrieve *f*_pv_ and *f*_npv_[7–9], which is preferred over traditional vegetation index methods. Conventional SMA approaches (i.e., Linear Spectral Mixture Model, LSMM) model a mixed spectrum as a linear combination of pure spectral signatures of its constituent components, weighted by their sub-pixel fractional cover[10]. LSMM has the advantage of yielding clear physical interpretations and easy computations. However, to what extent will the linear mixture assumption lead to the errors in *f*_pv_ and *f*_npv_ estimation, are the nonlinear mixture effects consistent among different desert vegetation types remains unclear, especially when the nonlinear mixing has been widely reported in plant-soil mixture [11–17]. Therefore, investigating the nonlinear mixing effects of typical desert vegetation types has great value for improving the *f*_pv_ and *f*_npv_ estimation accuracy.

Although, a few studies show that LSMMs tend to work well because plants are widely separated and thus the area of scattering is well localized covering a small area in sparsely vegetated arid regions[18, 19]. These results are not appropriate for retrieving PV and NPV separately for desert vegetation, where the vegetation canopy is open, PV and NPV grow closely together. Hence, the present study aims at investigating the nonlinear spectral mixture effects through comparing the performance of LSMM and Nonlinear Spectral Mixture Model (NSMM) for retrieving *f*_pv_ and *f*_npv_. Two types of dominant desert vegetation, *Nitraria* shrubs and *Haloxylon* in the transition zone of the Gansu Minqin Oasis, are chosen as the experiment desert vegetation. Unlike imagery-based studies, controlled in situ measurements allow the use of pixel- or plot-specific reference endmembers to minimize the effects of endmember variability.

## Data and methods

### Study area

**Fig 1:**
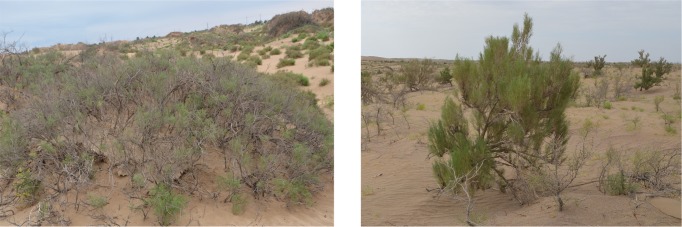
Typical desert vegetation. (left) *Nitraria* shrubs. (right) *Haloxylon*.

The study area is located the transitional zone (38°37'42.60"N, 102°55'11.25"E) between the oasis and the desert in the western region of Minqin County in the Gansu province along the downstream portion of the Shiyang River. This region consists of temperate continental arid climate zones, and the natural vegetation is mainly desert vegetation. A key characteristic of the desert vegetation is that it contains few species, and only a few layers are present with a simple structure and low productivity. *Nitraria* is short (1–2 m in the mature stage) with an open canopy structure, and it is a type of shrub [20, 21], as shown in Fig 1. *Nitraria* shrubs are relatively resistant to sandy and salty conditions and serve as windbreaks across the landscape. These shrubs form a natural ecological barrier in oasis environments in the arid region of western China [22]. *Haloxylon* is tall (1–9 m in the mature stage) with a compact canopy structure, and it is a xeric adapted plant [23, 24](Fig 1). Because of its deep roots, numerous branches, resilience, and adaptability, it is drought resistant and capable of growing in barren soil and areas with invasive sands; hence, it is a very valuable plant resource in arid desert regions. *Haloxylon* is the most common species used in artificial afforestation projects in the western area of the Gansu River basin [25].

### Spectral data and preprocessing

**Fig 2:**
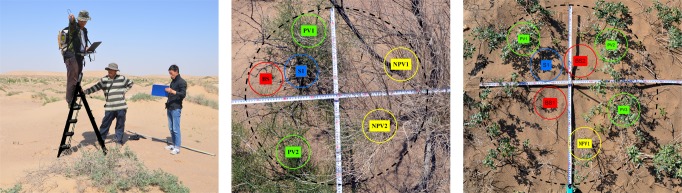
Processing of each endmember and mixed canopy spectral data collection in situ. (left) Acquisition of spectral data. (center) Sample selection for Nitraria shrub spectral data. (right) Sample selection for Haloxylon spectral data. PV represents the sunlit and shaded green leaves; NPV represents the sunlit and shaded wood, senescent material, and litter; BS represents the bare soil; shadow represents only the shade on the soil. The individual in this manuscript has given written informed consent (as outlined in PLOS consent form) to publish these case details.

In order to remove the effects of endmember variability, the experimental setup was designed so that endmembers were allowed to vary among plots. For each of the experimental plots, plot-specific bare soil (BS), shadow, PV, and NPV endmembers were defined and used in further analyses. In this research, spectra data for each endmember and canopy mixed spectral were obtained from ground-based field experiments; this involved obtaining specific PV/NPV/BS/shadow endmember spectra in each sample plot so that we could remove the effect of variability among the endmember spectra [11]. Reflectance spectral measurements were acquired on August 25, 2014 (a clear-sky day), within 1 h of local solar noon by using a full-range (350–2500 nm) spectroradiometer with a 25° ASD (Analytic Spectral Devices, Boulder, CO) Spec Pro Field spectrometer. The reflectance was calibrated by using a white spectral panel (Labsphere Inc., North Sutton, NH). During windless, cloudless, and full sunshine conditions, we collected data throughout a stable time period (10:00–14:00) from 20 *Nitraria* plots and 20 *Haloxylon* plots with different ratios of PV to NPV cover, and we measured the canopy spectra and the pure endmembers spectra, as shown in Fig 2. We used an orthogonal ruler to determine the center of the plots, and we marked out 1 m diameter circular area to ensure the sample range was consistent. Measurements were taken from nadir at a height of 2.3 m above the earth’s surface, which resulted in a field of view (FOV) or a pixel/plot diameter of 1 m from which we obtained the mixed spectral data for the fields, as illustrated in Fig 2; meanwhile, we placed the probe above the various typical species endmember surfaces (e.g., *Nitraria*, *Haloxylon*, dry branches, fine sand, shadows) between 0.1 m and 0.02 m so that pure endmember spectra were acquired in each field, as illustrated in Fig 2. In this way, each mixed canopy pixel and the specific pure endmember spectra for the field sites were obtained.

### Reference fraction

**Fig 3:**
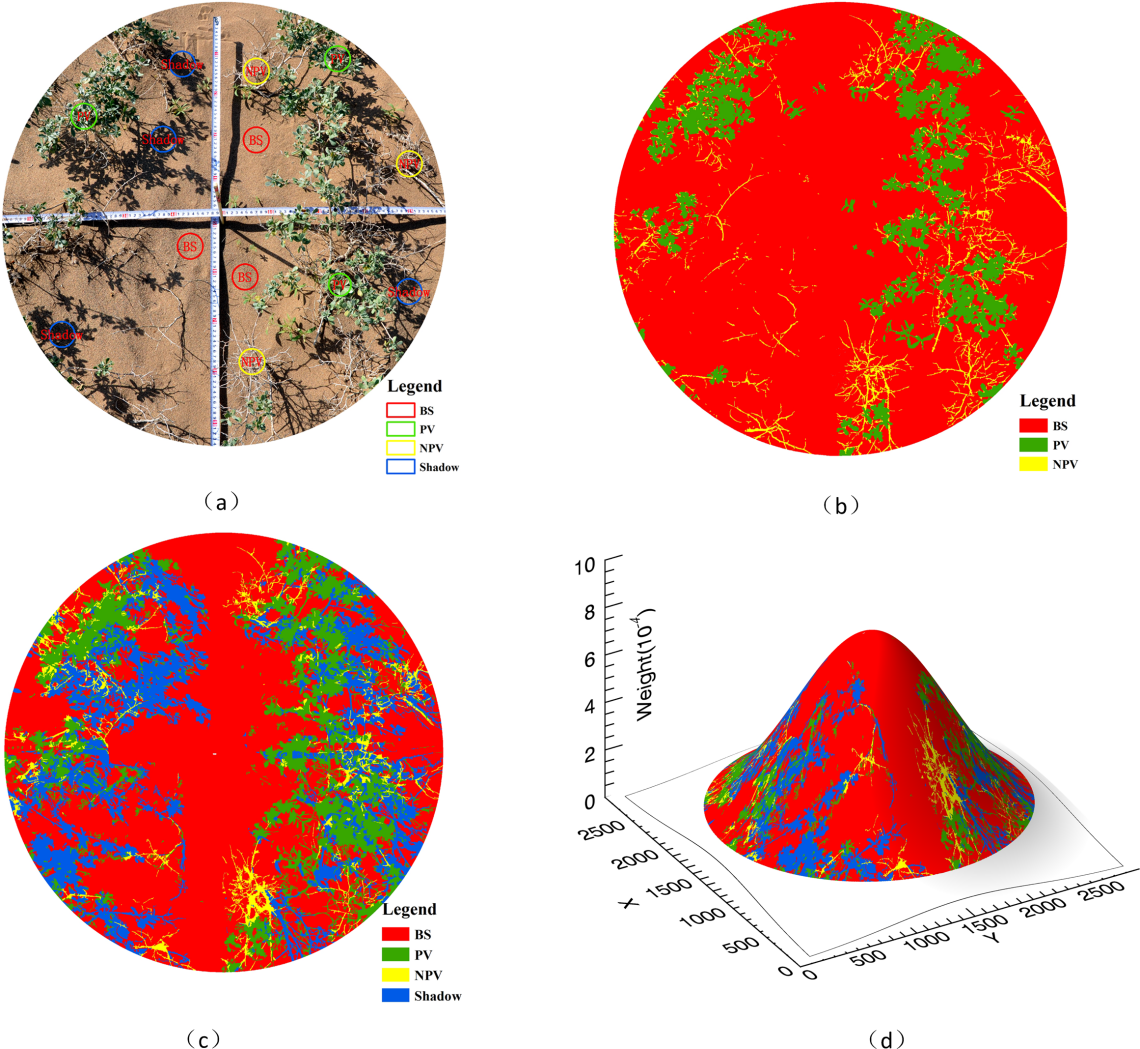
NNC classification for 3-EM and 4-EM image and conversion techniques with the reference fractional cover. (a) Selected training sample. (b) Classified origin image for 3-EM. (c) Classified origin image for 4-EM. (d) 3D simulation of the Gaussian spread function applied on the classified images to give a representative weight corresponding to the pixel reflectance as measured by the spectral sensor. The weights sum to 1.

**Fig 4:**
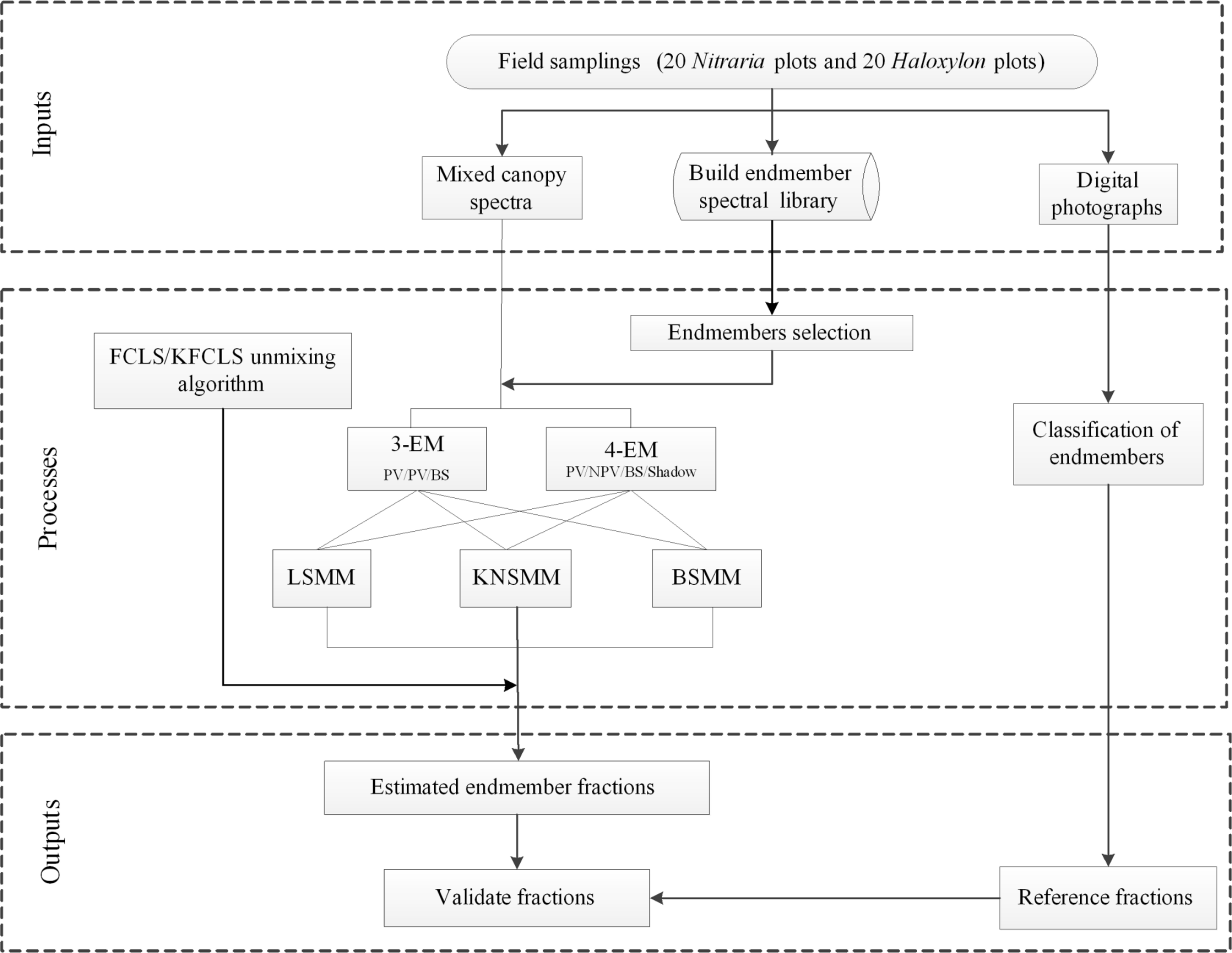
Methods flowchart showing the processing for the spectral mixture analysis and validation.

In this study, photographs were acquired two times by using a digital camera at each field site, in order to avoid out of focus. The photograph with higher quality will be selected, so that the photograph could be used to get the reference fraction accurately. Information on the ground cover composition of each of the measured mixed pixels was extracted from the digital photographs (positioned at nadir) so that the ground cover fraction distribution could be determined. As shown in Fig 3A, two cross rulers were used to mark the FOV while the digital image sensor was used to obtain RGB (red, green, and blue) photographs. According to the training samples for supervised classification, neural network classification (NNC) was applied to classify the digital photos with ENVI 5.3 software[26], and this yielded the 3-EM classes (Fig 3B) and 4-EM classes (Fig 3C). The classification results were validated through visual interpretations. The observed endmember cover would not contribute equally to the ASD probe due to the point spread function (PSF) of the ASD sensor. Hence, it was necessary to calibrate the fractional cover.

The PSF is the Gaussian function, $PSF(d)=\exp(-\frac{d^{2}}{2\sigma^{2}})$, where *d* is the distance from a pixel center (the unit is in meters) and σ illustrates the instantaneous FOV (IFOV) of a detector, which means that measurements closer to the center of the FOV will contribute more to the mixed reflectance signal, and thus, more weight should be given to the objects in the center. Each binary classified image was convolved with a Gaussian filter (i.e., *PSF* of the ASD sensor) to compute weighted averages of the image as shown in the Fig 3D. Endmember reference fractions could as such be expressed as a function of their actual contribution to the mixed pixel reflectance. Extensive research on this issue has been conducted by Settle [27] and Somers [28]. According to the PSF model, σ is 0.4363, which corresponds to a 25° IFOV for the ASD transformed to radians. Here, all the actual ground cover fractions were corrected to correspond to the PSF of the ASD sensor. In summary, all the classification images were convolved with a Gaussian filter, and then, the weighted average adjusted fractions of two photographs were taken as the reference fraction for the different endmembers in a plot.

## Spectral mixture models

In this case study, the methodological scheme is shown in Fig 4 and the detailed steps are followed in the sections.

### Linear spectral mixture model

In theory, linear spectral mixture modeling makes the physical assumption that each incident photon interacts with one earth surface component only, so the collected reflection spectra do not mix (i.e., no multiple scattering) before entering the sensor [29, 30]. We have constructed the LSMM (Eq (1)) based on the principles of linear mixtures[13, 31–36]. The mixed spectra of the LSMM can be regarded as the linear combination of the endmember spectral response. In its general form, the LSMM can be described as follows:

$$R_{i}=\sum_{j=1}^{m}\left(f_{j}W_{i,j}\right)+\varepsilon_{i}$$ (1)

where *R*_*i*_ is the measured reflectance of a mixed pixel in spectral band *i*; *f*_*j*_ is the sub-pixel cover fraction of the *j*th endmember in the pixel; and *W*_*i*,*j*_ is the *j*th endmember reflectance for spectral band *i*; *m* is the number of the endmembers. Based on the measured pixel spectral vector R and the endmembers’ spectral vector W, and this is done under the constraints that *f*_*j*_ ≥ 0 for j = 1, …, m (ANC) and $\sum_{j=1}^{m}f_{j}=1$ (ASC). Since every pixel spectral, R, is acquired by spectral channels at different wavelengths, it can be represented by a column vector of which each component is a pixel in a plot. By making use of Eq (1), the endmember fraction *f*_*j*_ estimates are obtained by the fully constrained least squares (FCLS) [37, 38], for which the following equation is minimized:

$$min\sum_{i=1}^{N}{\left\|\sum_{j=1}^{m}\left(f_{j}W_{i,j}\right)-R_{i}\right\|}^{2}$$ (2)

where *n* is the number of effective spectral bands; and. *ε*_*i*_ is the spectral model error. To impose the ASC, the linear mixing model is written as follows:

$$M=\left[\begin{array}{c} \delta W\\ 1^{T} \end{array}\right]$$ (3)

$$with\;1={\underset{m}{\underset{︸}{\left(1,1,\ldots ,1\right)}}}^{T},\;and\;a\;vector\;F\;by\;F=\left[\begin{array}{c} \delta R\\ 1^{} \end{array}\right]$$ (4)

Here,δ is a parameter that weights the strength of the sum to one constraint; *m* is the number of the endmember.

The non-negatively constrained least squares (NCLS) impose the ANC on the abundance vector. The iteration algorithm proposed in [38] was adopted by introducing a Lagrange multiplier vector (λ) in Eqs (5) and (6) to generate the solution:

$${\hat{f}}_{FCLS}={\left(M^{T}M\right)}^{-1}M^{T}F-{\left(M^{T}M\right)}^{-1}\lambda$$ (5)

$$\lambda =M^{T}(F-M{\hat{f}}_{FCLS})$$ (6)

#### Nonlinear spectral mixture model

According to the characteristics of different NSMMs [39], this study proposes the use of a bilinear spectral mixture model (BSMM), which is relatively simple to use and yields results with physical meaning, and a kernel-based NSMM.

#### Bilinear spectral mixture model

The bilinear spectral mixture models account for the presence of multiple photon interactions by introducing additional “interaction virtual” terms in the LSMM. Each term accounts for multiple interactions between endmembers and is represented by the cross-product of the interacting endmembers. BSMMs consider multi-order interactions between endmembers *j* and *t* (for *j*,*t* = 1,…,m). Popular BSMMs include the Fan Model (FM)[40], Generalized Bilinear Model (GBM) [41], and Nascimento Model [42]. Considering the characteristics of *Nitraria* shrubs and *Haloxylon* structure in the study, we elected to use the Nascimento Model (Eq (7)) without considering higher-order multiple scattering, i.e., we only considered the second order between endmembers and PV/NPV themselves as scattering.

$$R_{i}=\sum_{j=1}^{m}\left(f_{j}W_{i,j}\right)+\sum_{j=1}^{m}\sum_{t=1}^{m}\left(f_{j,t}W_{i,j}W_{i,t}\right)+\varepsilon_{i}$$ (7)

where *R*_*i*_ is the measured reflectance of a mixed pixel in spectral band *i*, *f*_*j*_ is the sub-pixel cover fraction of the *j*th endmember in the pixel, *W*_*i*,*j*_ is the *j*th endmember reflectance for spectral band *i*, *W*_*i*,*j*_*W*_*i*,*t*_ denotes the nonlinear combination of multiple scattering effects between endmembers, and *ε*_*i*_ is the spectral model error, *f*_*j*,*t*_ describes the fraction of the *t*th second order mixture effects involving endmember *j*. The number of fraction products *m* is determined by that of the selected physical endmembers, and the number of fractions estimated includes all physical and virtual endmembers. By making ASC (sum to one constraint) and ANC (non-negativity constraint) constraints in the mixture model, and when ∀ *j ≥ t*, then *f*_*j*,*t*_ = 0, and when ∀ *j < t*, then *f*_*j*,*t*_ *≥* 0; meanwhile:

$$f_{j}\geq 0\;and\;\sum_{j=1}^{m}f_{j}+\sum_{j=1}^{m-1}\sum_{t=j+1}^{m}f_{j,t}=1$$ (8)

In Eq (8), the virtual multiple scattering term is applied as additional endmembers. The FCLS algorithm is employed to unmixing *f*_*j*,*t*_ and *f*_*j*_, so *f*_*j*,*t*_ is not related to *f*_*j*_, which means they are separate. Because of the sum to one constraint (Eq (8)) the ‘virtual’ fraction brings about significant underestimations of the actual ground cover, part of the interaction fraction *f*_*j*,*t*_ should be assigned to each of the contributing physical entities[28]. The cover fraction *f*_*j*_ can easily be isolated from *f*_*j*,*t*_ and estimated from Eq (9) as:

$$f_{j}^{}=f_{j}^{\left(1\right)}/\left(1-\sum_{t=1}^{n}f_{j,t}^{\left(2\right)}\right)$$ (9)

where *f*_*j*_ is the fraction of the first-order interaction of endmember *j*, $f_{j}^{\left(1\right)}$ is the single scattering fraction, $f_{j,t}^{\left(2\right)}$ describes the fraction of the *t*th second-order mixture effects involving endmember *j*, and *n* is the number of the endmembers. The reader is invited to consult [17], [40] and [42] for more details.

#### Kernel-based nonlinear spectral mixture model

**Kernel method.** The principle of the Kernel Nonlinear Spectral Mixture Model (KNSMM) is that the data from the input space *R*^*N*^ are mapped to the high-dimensional feature space *H*, through the implicit nonlinear mapping by kernel functions. By doing this, combinations of the original endmember spectral bands (i.e., some high-order multiplications of the original spectral bands) are now consisted of the components of each mapped endmember in the high-dimensional feature space. Therefore, the nonlinear mixture model is still linear and additive in the feature space but includes nonlinear components of the endmember spectral bands in the original input space [43–47].

Generally, the nonlinear mapping *ϕ* is unknown and may be complicated. Kernel-based learning algorithms use an effective kernel trick to implement dot products in feature space by employing some kernel functions[48]. The kernel representation for the dot products in *H* is expressed as:

$$K(x_{i},x_{j})=\langle \phi (x_{i}),\phi (x_{j})\rangle =\phi (x_{i})\cdot \phi (x_{j})$$ (10)

Then everywhere that *x*_*i*·_*x*_*j*_ occurs, we replace it with *K*(*x*_*i*_, *x*_*j*_). Theoretically, any function that satisfies the Mercer’s theorem[49, 50] or the Positive definite can be used as a kernel function. A Mercer Kernel is symmetric (*K*(*x*_*i*_, *x*_*j*_) = *K*(*x*_*j*_, *x*_*i*_)) and positive definite ((*K*(*x*_*i*_, *x*_*j*_) >0)). By Mercer’s theorem, any symmetric positive definite kernel represents the inner product in some higher-dimensional Hibert Space[50, 51]. According to the simple structure characteristics of the surface vegetation in the study area, we used two common kernel functions, namely, the radial basis function (RBF) kernel and the polynomial kernel function (PKF). The RBF and PKF were chosen due to their successful applications to non-linear unmixing in the scalar value case[46, 52]. RBF represents the case where an endless number of reflections occurs since it incorporates all higher order interactions between the input spectra, while PKF define the interactions order and has a relatively explicit physical meaning. The radial basis kernel function:

$$K(x_{i},x_{j})=\exp(-\frac{{\left\|x_{i}-x_{j}\right\|}^{2}}{2\sigma^{2}})$$ (11)

where σ is the parameter of the kernel function, and *x*_*i*_ and *x*_*j*_ are the spectral reflectance of endmember *i* and *j*. The polynomial kernel function:

$$K(x_{i},x_{j})={\left(ax_{i}{}^{T}x_{j}+c\right)}^{b}$$ (12)

where *a*, *b*, and *c* are the parameters of the kernel function.

**Parameters of kernel function.** The optimal parameters in the RBF and PKF are determined by the minimum model unmixing RMSE. Because the training sample data is different for the different models and the different types of vegetation, the optimal parameters value of the kernel function is different. The parameter σ in the RBF is determined by the gradient descent method [53], and it was determined to 200 with the four endmember (4-EM) models and to 20 with the three endmember (3-EM) models in the *Nitraria* shrubs plots, and to 152 with the 4-EM models and to 200 with the 3-EM models in the *Haloxylon* plots. The parameters of PKF are determined by the Cross validation [54–57] which means that the fitting process optimizes the model parameters to make the model fit the input data as well as possible. The training data are split into *k* parts of size l/*k*. A discrete range of possible values of the kernel functions parameter is chosen and for each parameter value a spectral unmixing is trained using *k*-1 parts and tested on the remaining 1 part from which a model unmixing accuracy is measured since we know the labels of the data. This is repeated one by one through all *k* such splits of the training data into *k*-1 folds for training with testing on the remaining fold, and an average model unmixing accuracy is obtained. This is repeated for each of the parameter values and the value with the highest model unmixing accuracy is chosen. The polynomial order b = 2 was determined in the experiment, which refers to fourth-order statistical properties of the spectral data. a = 1/*n*, where *n* is the number of endmembers, and c = 1.

**Kernel fully constrained least squares (KFCLS).** The KFCLS aims to find the abundance vectors ${\left\{f_{j}\right\}}_{j=1}^{m}$ via the objective function:

$$min\sum_{i=1}^{n}{\left\|\sum_{j=1}^{m}\left(f_{j}\phi (W_{i,j})\right)-\phi (R_{i})\right\|}^{2}$$ (13)

The KFCLS algorithm can be derived directly from the FCLS algorithm described in the previous section by replacing *M*^*T*^*M* and *M*^*T*^*F* used in the FCLS algorithm [44, 58]. Both of Eqs (5) and (6) are kernelized by

$${\hat{f}}_{KFCLS}={\left(K(M,M)\right)}^{-1}K(M,F)-{\left(K(M,M)\right)}^{-1}\lambda_{KFCLS}$$ (14)

$$\lambda_{KFCLS}=K(M,F)-K(M,M){\hat{f}}_{KFCLS})$$ (15)

It should be noted that *K*(*M*, *M*) and *K*(*M*, *F*)in Eqs (14) and (15) are a kernel version of *M*^*T*^*M* and *M*^*T*^*F*, respectively. A detailed step-by-step implementation of KFCLS is available in[58], [59] and [60].We refer its derivations to these reference.

### Methods for evaluating error

In cases where accurate ground reference data are available, the quality of the sub-pixel abundance estimates can be assessed more reliably by checking the discrepancy between the estimated and reference endmember fractions. The spectral mixture model fit was checked by using the unmixing error of the spectral mixture model, the PV/NPV/BS/shadow ground validation RMSE (Eq (16))[40], the *R*^2^ (Eq (17)) [7], the significance *p*-value and the Relative RMSE (RRMSE) (Eq (18))[61]. The use of the RMSE of the spectral mixture model is mainly aimed at validating the accuracy of the model in unmixing the mixture spectral. The RMSE of endmembers was calculated to quantify the difference between the measured fraction and estimated fraction for the fields. The *p*-value is the probability for a given statistical model and represents the significance level in the statistical hypothesis testing, which can calculated by statistical software (e.g. SPSS or SAS). The smaller the *p*-value, the larger the significance. RRMSE is a measure of the deviation rate with percent as the unit, and values closer to zero are indicative of a better fit. The relevant equations are as follows:

$$RMSE=\sqrt{\sum_{i=1}^{n}{\left(x_{i}-y_{i}\right)}^{2}/n}$$ (16)

$$R^{2}=\frac{(\sum {}_{i=1}^{n}(x_{i}-\bar{x})(y_{i}-\bar{y}){)}^{2}}{\sum {}_{i=1}^{n}{\left(x_{i}-\bar{x}\right)}^{2}\sum {}_{i=1}^{n}{\left(y_{i}-\bar{y}\right)}^{2}}$$ (17)

$$RRMSE=\sqrt{\left(\sum_{i=1}^{n}{\left(x_{i}-y_{i}\right)}^{2}/n\right)}/\bar{y}\;*\;100\%$$ (18)

where RMSE is the root mean square error, *R*^2^ is the square of the correlation coefficient, RRMSE is Relative RMSE of the estimated model spectral value or estimated cover fraction of endmembers, *n* is the number of fields or available wavebands, *x*_*i*_ is the estimated cover fraction or the estimated mixing spectral value of the *i*th field, *y*_*i*_ is the measured cover fraction or measured mixing spectral value of the *i*th field, $\bar{x}$ is the average value of the estimated cover fractions, and $\bar{y}$ is the average value of the measured cover fractions.

Describe the same contents as “Data and Methods” and “Spectral Mixture Models” sections with step-by-step protocol on my protocols.io: http://dx.doi.org/10.17504/protocols.io.ia5cag6.

## Results

### Spectral characteristics of endmembers

In order to achieve the estimation of *f*_pv_ and *f*_npv_, the mixed canopy spectra and PV/NPV/BS/shadow spectra of each plot were obtained by ground spectrum observations. Considering the applicability and representativeness of endmembers, the severely affected bands and water vapor absorption in the atmospheric bands were removed and we kept the spectral range of 350–1350 nm, 1450–1750 nm, and 2000–2350 nm [62]. In order to remove the influence of the endmember variability in relation to the temporal and spatial data, the average value of five sets of measured spectra for each endmember was adopted as the real endmember spectra of PV/NPV/BS/shadow in a field.

**Fig 5:**
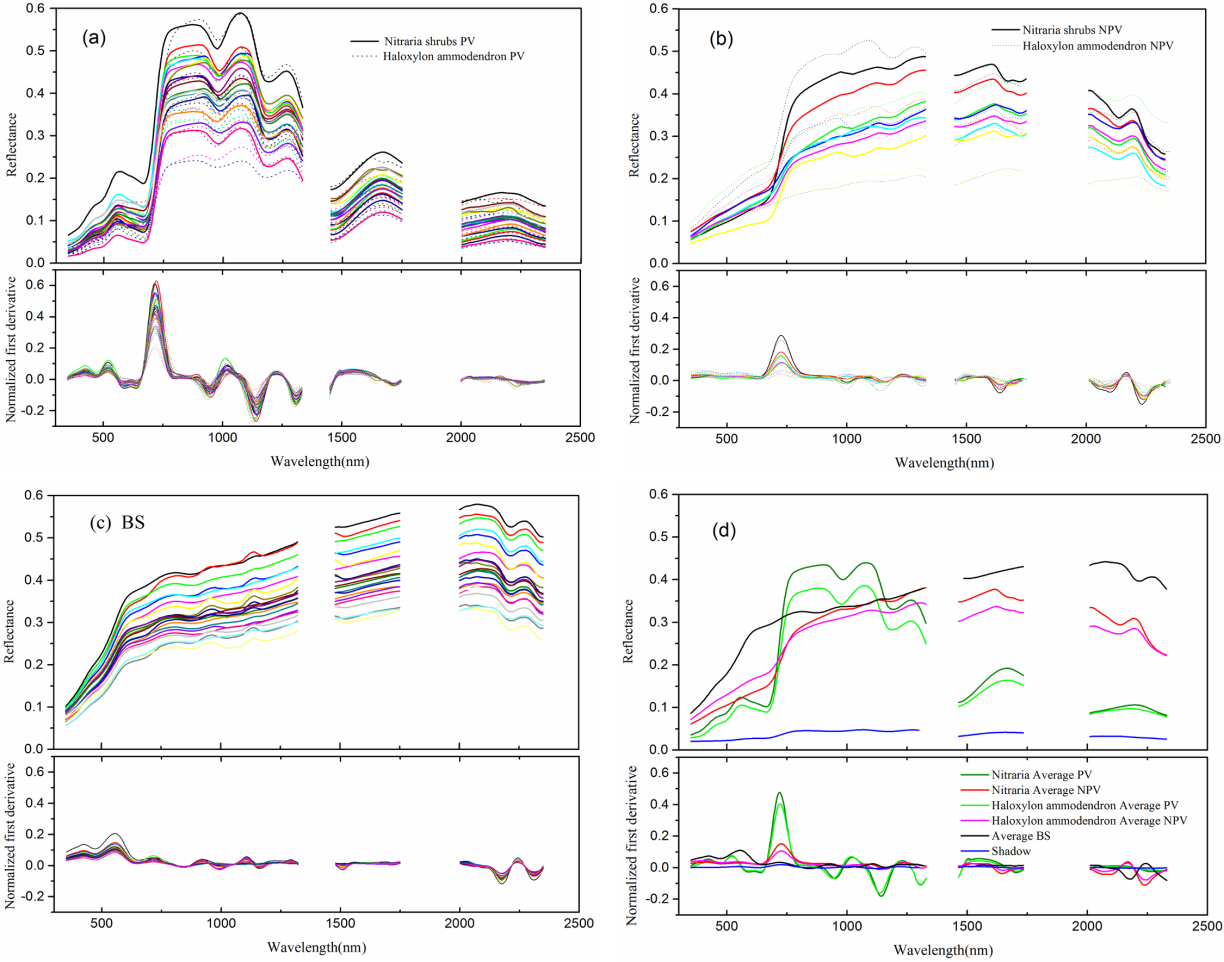
The spectral library accompanied by the normalized first derivative graphs of each endmember class from *Nitraria* shrubs and *Haloxylon* fields. (a) Photosynthetic vegetation (PV); (b) non-photosynthetic vegetation (NPV); (c) bare soil (BS); (d) the average spectra of each endmember class in the study. The normalized first derivative for all endmembers spectra have been magnified 100 times.

As shown in Fig 5, the results illustrate the spatial variability of the endmember spectra collected and their normalized first derivative graphs indicating the slope of the wavelength vs. reflectance in the different plots. Fig 5A shows that the actual spectral characteristics of the PV endmember among pixels were obviously different from the range of 750–1250 nm, while the major variability spectral range of the BS endmember covered the 750–2350 nm spectral domain (Fig 5C). Especially, there were obvious differences among the spectral curves of the NPV endmember (Fig 5B) in the different grown stages. Due to the diverse endmember forms, the endmember libraries show large intra-variability (Fig 5A–5C) at the full wavelength range. In Fig 5D, the results show the average spectral curves of the different endmembers. Through the whole spectral curve, it can be seen that PV display obvious differences in reflectance between red and near infrared reflectance, while the reflectance of NPV and BS did not have this feature; consequently, PV was relatively easy to distinguish from NPV and BS. However, the NPV and BS spectra curves were very similar, and thus, it was difficult to distinguish them. Despite this issue, the spectra curve of the NPV endmember was different from BS in two narrow ranges, namely, 500–900 nm and around 2100 nm. In particular, there was an obvious bow shaped protuberance area between the 500 nm and 900 nm spectral range with the BS endmember[63]. The non-cellulose structure component of NPV caused the absorption features around 2100 nm [64]. The reflectivity of shadows was low and little variability was observed over the whole spectral range; the shadows showed significant differences from the other types of endmembers. According to the normalized first derivative graphs of the endmembers spectra, red edge (670–760 nm) is the most obvious feature of the first derivative curve of PV, then it is NPV, therefore, red edge is a goodness spectral domain for PV and NPV distinguishing from BS and shadows.

### Effects of endmember selection

Through choosing two endmember combinations, 3-EM and 4-EM respectively, LSMM and NSMM were adopted to retrieve *f*_pv_ and *f*_npv_ for s *Nitraria* shrubs and *Haloxylon* plots. In the BSMM, according to the vegetation structure characteristics of *Nitraria* shrubs and *Haloxylon*, 286 sets of BSMMs including different virtual endmembers (Eqs (7–9)) were designed, in which, 31 sets based on the 3-EM models and 255 sets based on the 4-EM models. Then, we calculated the fractional cover of each endmember with the field data by the FCLS (Eqs (3–6)) and KFCLS (Eqs (14 and 15)) techniques. The estimated fractional covers were verified by the real measured fractional covers in the field according to Eqs (16–18). The results are summarized and illustrated in Table 1, wherein the data include five BSMM results for the best subset selections with high accuracy in the 3-EM and 4-EM models.

**Table 1:** RMSE, R^2^, p-value, and relative RMSE for the LSMM and NSMM extracted data and reference sub-pixel cover fractions of *Nitraria* shrubs and *Haloxylon*.

Models		*Nitraria* shrubs										*Haloxylon*									
		MODEL RMSE	RMSE (%)	R^2^				Relative RMSE (%)				MODEL RMSE	RMSE (%)	R^2^				Relative RMSE (%)			
				PV	NPV	BS	Shadow	PV	NPV	BS	Shadow			PV	NPV	BS	Shadow	PV	NPV	BS	Shadow
**PV-NPV-BS mixtures**																					
**LSMM**		**0.0640**	**28.47**	0.15***	0.65***	0.52***	**—**	121.09	83.86	17.54	—	**0.0523**	**23.22**	0.58***	0.71***	0.45***	**—**	98.08	75.65	42.06	
**NSMM**																					
KNSMM	KNSMM_RBF	**0.0430**	**18.32**	0.05^ns^	0.01^ns^	0.06^ns^	**—**	166.69	473.82	69.44	—	**0.0332**	**16.00**	0.68***	0.21*	0.17^ns^	**—**	76.04	195.87	62.00	—
	KNSMM_PKF	**0.0429**	**18.31**	0.11^ns^	0.01^ns^	0.06^ns^	**—**	153.71	450.11	64.45	—	**0.0333**	**16.02**	0.69***	0.21*	0.18^ns^	**—**	75.71	195.62	62.07	—
BSMM	3-EM+P^2^	**0.0414**	**17.03**	0.15^ns^	0.64***	0.57***	**—**	90.26	93.41	12.53	—	**0.0337**	**13.69**	0.69***	0.48***	0.43***	**—**	98.08	75.65	42.06	—
	3-EM+P*B	**0.0228**	**9.09**	0.10^ns^	0.65***	0.49***	**—**	99.25	75.48	13.19	—	**0.0277**	**10.86**	0.60***	0.76***	0.55***	**—**	96.03	67.20	36.28	—
	3-EM+B*N+P*B	**0.0182**	**7.43**	0.07^ns^	0.06*	0.27^ns^	**—**	151.53	114.80	17.21	—	**0.0245**	**9.92**	0.60***	0.76***	0.53***	**—**	95.83	60.17	33.58	—
	3-EM+P^2^+N^2^+P*B	**0.0151**	**5.98**	0.16^ns^	0.07*	0.24^ns^	**—**	108.90	119.63	17.32	—	**0.0224**	**9.36**	0.70***	0.78***	0.66***	—	76.14	70.22	32.28	—
	3-EM+P^2^+N^2^+P*N+B*N+P*B	**0.0154**	**6.01**	0.24*	0.10^ns^	0.36**	**—**	85.67	112.71	15.97	—	**0.0359**	**15.06**	0.54***	0.44***	0.53***	**—**	107.06	78.24	38.53	—
**PV–NPV–BS–shadow mixtures**																					
**LSMM**		**0.0069**	**2.83**	0.20^ns^	0.64***	0.44***	0.29*	76.84	62.31	28.90	89.50	**0.0133**	**6.25**	0.75***	0.82***	0.21*	0.01^ns^	54.98	43.92	54.13	204.66
**NSMM**																					
KNSMM	KNSMM_RBF	**0.0052**	**2.16**	0.19^ns^	0.66**	0.42***	0.30*	79.65	61.42	29.69	87.03	**0.0079**	**3.88**	0.79***	0.79***	0.25*	0.00^ns^	48.71	43.89	51.60	195.41
	KNSMM_PKF	**0.0067**	**2.81**	0.19^ns^	0.66**	0.40***	0.24*	79.75	60.97	31.74	91.41	**0.0089**	**4.14**	0.84***	0.78***	0.26*	0.00^ns^	44.14	59.20	52.62	192.24
BSMM	4-EM+P^2^	**0.0069**	**2.81**	0.18^ns^	0.63***	0.43***	0.28*	78.55	63.02	28.82	89.11	**0.0124**	**5.19**	0.78***	0.81***	0.21*	0.00^ns^	47.04	43.72	53.93	209.19
	4-EM+P*S+B*N	**0.0070**	**2.87**	0.19^ns^	0.54***	0.38***	0.13^ns^	78.19	68.64	29.52	91.10	**0.0109**	**4.95**	0.58***	0.84***	0.21*	0.04^ns^	85.91	43.18	56.51	212.57
	4-EM+N^2^+B*S+N*S	**0.0064**	**2.62**	0.24*	0.46**	0.33**	0.06^ns^	73.06	80.87	30.14	92.16	**0.0107**	**4.93**	0.61***	0.64***	0.22*	0.04^ns^	83.84	58.15	52.18	211.17
	4-EM+N^2^+P*N+P*S+B*S+N*S	**0.0056**	**2.31**	0.18^ns^	0.59***	0.20***	0.00^ns^	82.22	72.10	35.19	100.74	**0.0091**	**4.31**	0.59***	0.87***	0.26*	0.04^ns^	88.77	37.10	53.03	197.33
	4-EM+P^2^+N^2^+P*B+P*N+P*S+B*S+N*S	**0.0061**	**2.49**	0.18^ns^	0.44**	0.34**	0.24*	79.17	82.53	31.24	87.02	**0.0084**	**4.12**	0.58***	0.67***	0.30*	0.02^ns^	92.49	61.49	50.17	179.90

P = PV, B = BS, N = NPV, S = shadow.

*** *p*<0.001.

** *p*<0.01.

* *p*<0.05.

ns: not significant.

As shown in Table 1, regardless of whether the LSMM or NSMM was used, there was a high RMSE when estimating the fractional cover by the traditional PV-NPV-BS 3-EM. The model RMSEs for *Nitraria* shrubs and *Haloxylon* were 0.064 and 0.0523 in the LSMM, respectively. When the shadow endmember was introduced, for both the *Nitraria* and *Haloxylon* plots, the accuracy of the 4-EM models was much better than that of the 3-EM models. The model unmixing absolute RMSE decreased to 0.0069 and the model relative RMSE decreased to 2.83% with the LSMM in the *Nitraria* shrub plots; meanwhile, these values decreased to 0.0133 and 6.25%, respectively, in the *Haloxylon* plots. In both the *Nitraria* and *Haloxylon* plots, all absolute RMSEs of the 4-EM models and relative RMSE% values were lower than those of the 3-EM models with the NSMM. From the model RMSEs for the LSMM and NSMM, we can conclude that the 4-EM models are more suitable to use to unmix the *Nitraria* shrubs and *Haloxylon* canopy mixed spectra.

**Fig 6:**
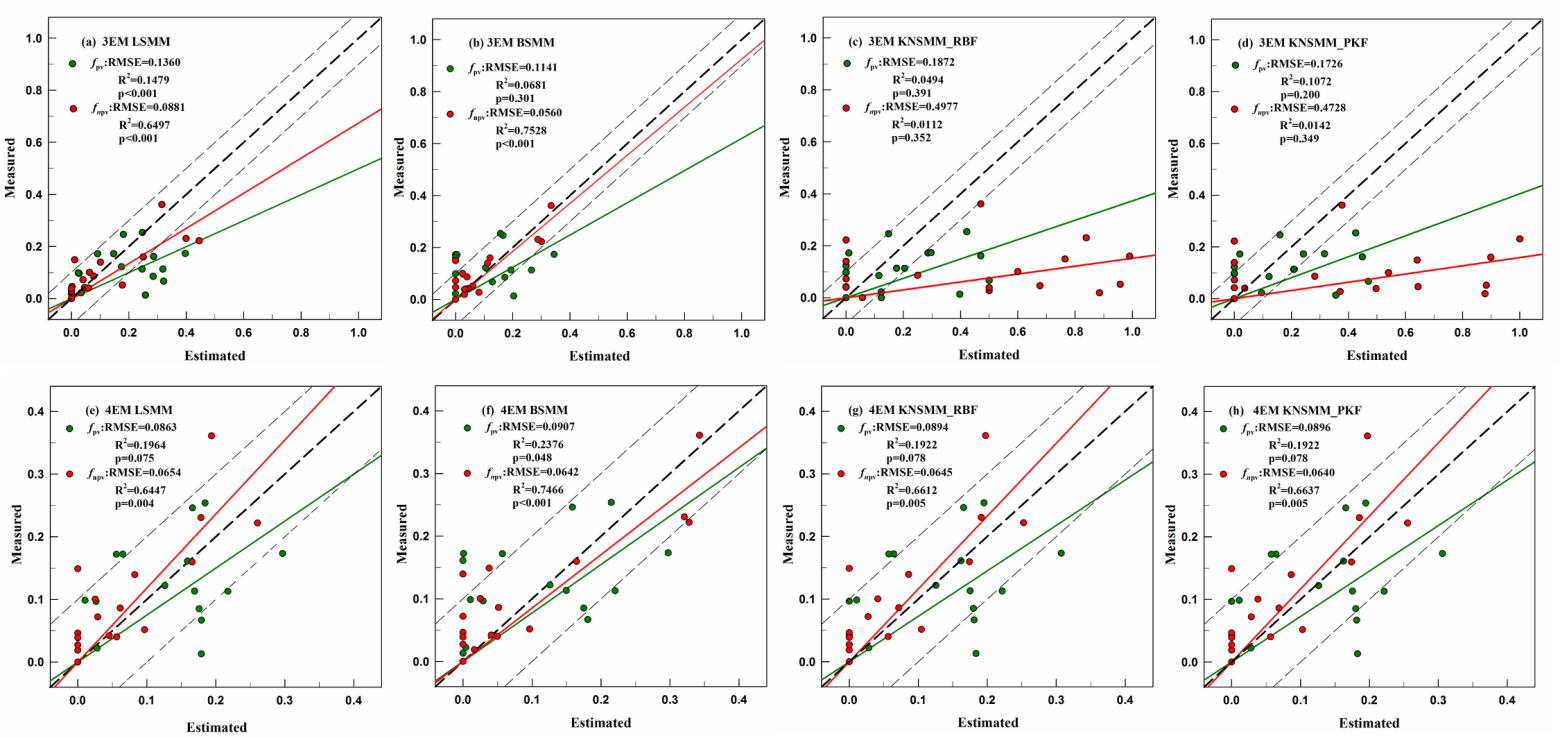
Scatterplots and the best fit regression lines of the estimated and measured *f*_pv_ and *f*_npv_ from the LSMM and NSMMs based on 3-EM and 4-EM in the *Nitraria* shrubs plots. The diagonal line corresponds to the 1:1 agreement, and the dotted lines indicate a ±10% deviation. N = 17 in all scenarios.

**Fig 7:**
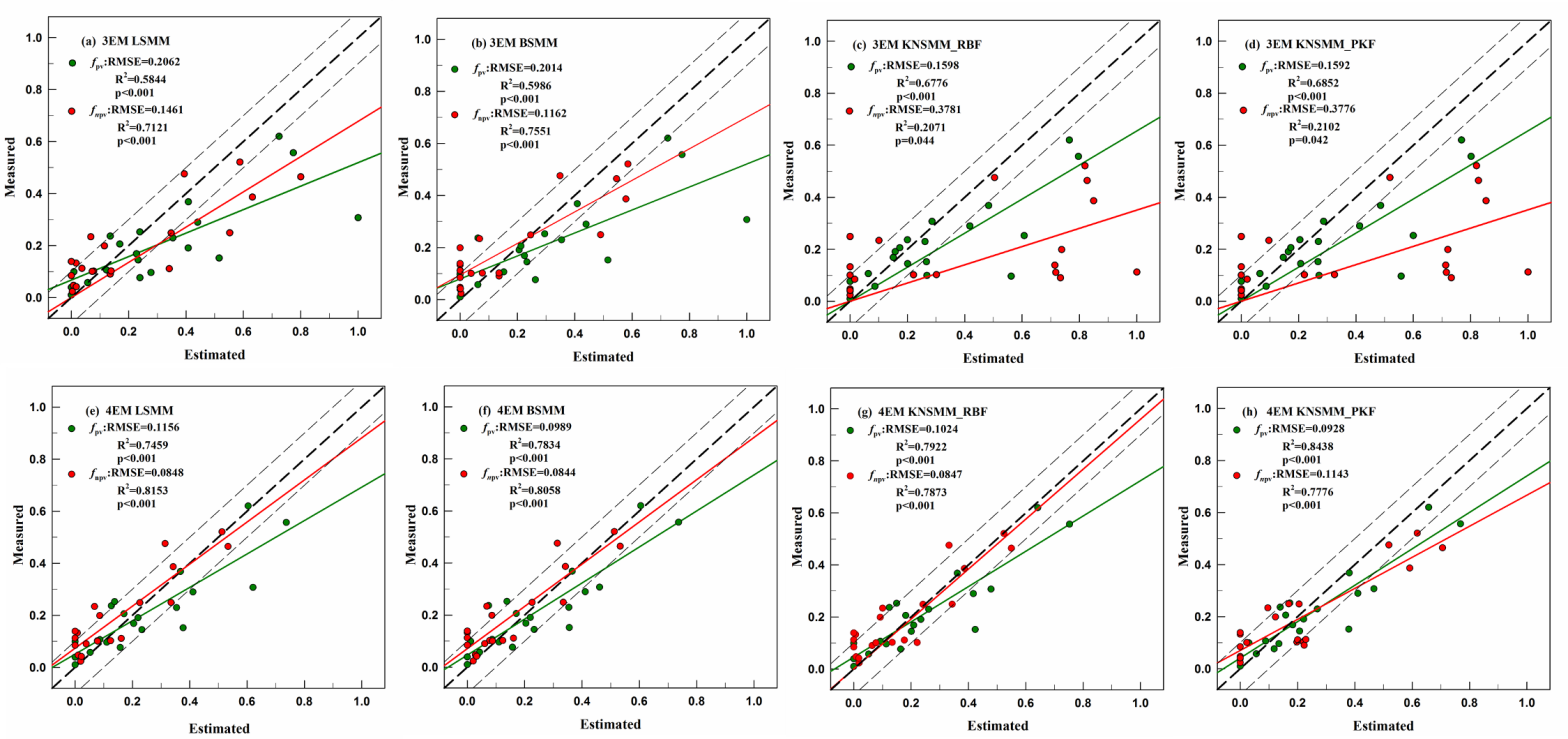
Scatterplots and the best fit regression lines of the estimated and measured *f*_pv_ and *f*_npv_ from the LSMM and NSMMs based on 3-EM and 4-EM in the *Haloxylon* plots. N = 20 in all scenarios.

**Fig 8:**
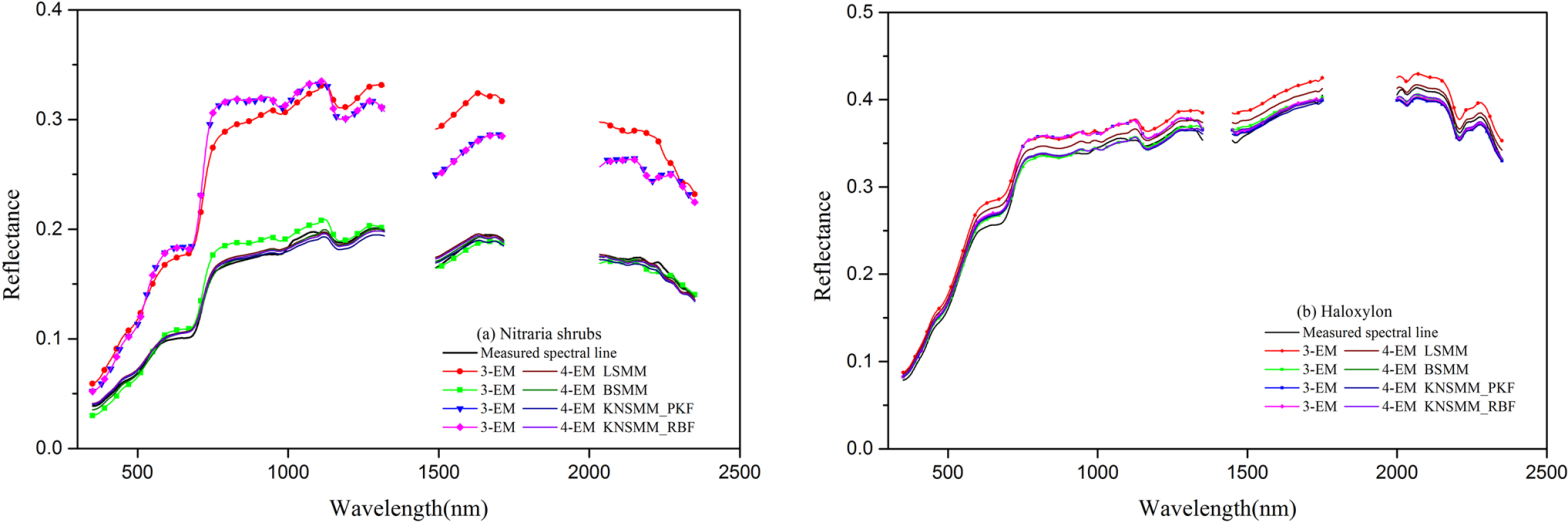
Comparison of the simulated mixed canopy spectral lines to the measured spectral line. (left) *Nitraria* shrub plots. (right) *Haloxylon* plots.

We drew scatterplots and the best fit regression lines of the estimated and measured PV and NPV fractional cover in the *Nitraria* shrubs (Fig 6) and *Haloxylon* (Fig 7) plots. Through using the LSMM, when the 4-EM models were used instead of the 3-EM models, the RMSEs of *f*_pv_ and *f*_npv_ estimation were significantly reduced, the relative RMSE% of *f*_pv_ and *f*_npv_ decreased by 44.25% and 21.55% for the *Nitraria* shrubs plots, 43.01% and 31.73% for the *Haloxylon* plots. When the BSMMs and KNSMMs were used, the accuracy of the 3-EM models was slightly improved compared to those of the LSMM, but the 3-EM models did not exceed the performance of the 4-EM models. Compared with the 3-EM models, the slopes of the best fit regression line of the 4-EM models were obviously closer to the 1:1 line and more points were located in the ±10% dotted lines for both vegetation types, and a stronger correlation between the estimated and measured fractional cover for all endmember was obtained with the 4-EM models, especially for the NPV endmember. The overestimated fractional covers obtained with the 3-EM models were corrected through using the 4-EM models. Regardless of whether *Nitraria* shrub plots or *Haloxylon* plots were tested, it was found that the 4-EM models were more efficient than the 3-EM models for estimating the fractional cover of *f*_pv_ and *f*_npv_. In Fig 8, the data show typical calculated spectral line simulations for the LSMMs, BSMMs, and KNSMMs based on the 3-EM and 4-EM approaches and the measured canopy spectral lines in the plots. We also found that the canopy mixed pixel simulated spectral lines by the 4-EM models were closer to the measured pixel spectral line with the same characteristics in both graphs.

Above all, compared with the performance of 3-EM models to estimate *f*_pv_ and *f*_npv_, the performance of the 4-EM models was obviously better, and the same conclusion was drawn for the *Nitraria* shrub plots and *Haloxylon* plots. The results also demonstrate that the shadow endmember is not negligible in *Haloxylon* and *Nitraria* shrubs fields. Therefore, the rationality of the selection of the endmembers can play a significant role in improving the accuracy of *f*_pv_ and *f*_npv_ estimations.

### Nonlinear spectral mixture effects analysis

Nonlinear spectral mixture effects were investigated through comparing the performance of the LSMM, BSMM, and KNSMM techniques with the 4-EM models. For the BSMM models, when the virtual interactive multiple photon scattering terms between PV or NPV and BS or shadow endmembers were added in the models, the precision of *f*_pv_ and *f*_npv_ estimation was improved. Compared to the LSMM, the RMSEs of the BSMM for unmixing decreased from 0.0133 to 0.0084 for *Haloxylon* plots and from 0.0069 to 0.0056 for *Nitraria* shrub plots, and their RMSE% values were reduced by 0.52% and 2.13%, respectively. The effect of self-high-order scattering was not obvious in *Nitraria* shrubs plots, while it was slightly obvious in the *Haloxylon* plots. In addition, when the endmember fractional covers were too low, it was easy to cause their estimated fraction value to be zero with the BSMM; therefore, caution is necessary when using this approach.

With the KNSMM, the model unmixing RMSE and the fractional cover estimation RMSE of the endmembers were lower than those of the LSMM and BSMM for both vegetation types. Especially for the Haloxylon plots, the model unmixing RMSE decreased from 0.0133 to 0.0079, while the relative RMSE% decreased from 6.25% to 3.88% when the KNSMM was used. Regardless of the RBF or PKF kernel function, there was a stronger correlation between the estimated and measured fractional cover of each endmember in the 4-EM models with the KNSMM than those in the LSMM and BSMM. The RBF kernel function can deal with the complexity of space well, and the PKF kernel function is useful for illustrating the physical meaning of the space and the high-order statistical properties of the inter bands. For the effects of different kernel, the RBF kernel function with the NSMM was more reliable than the PKF kernel function with the NSMM for vegetation cover estimations both in the *Nitraria* shrubs plots (ΔRMSE_RBF-PKF_ = -0.0012, ΔRRMSE% _RBF-PKF_ = -0.65%) and *Haloxylon* plots (ΔRMSE_RBF-PKF_ = -0.001, ΔRRMSE% _RBF-PKF_ = -0.26%). In summary, the KNSMM considering the nonlinear parameters was better than the LSMM and BSMM.

**Fig 9:**
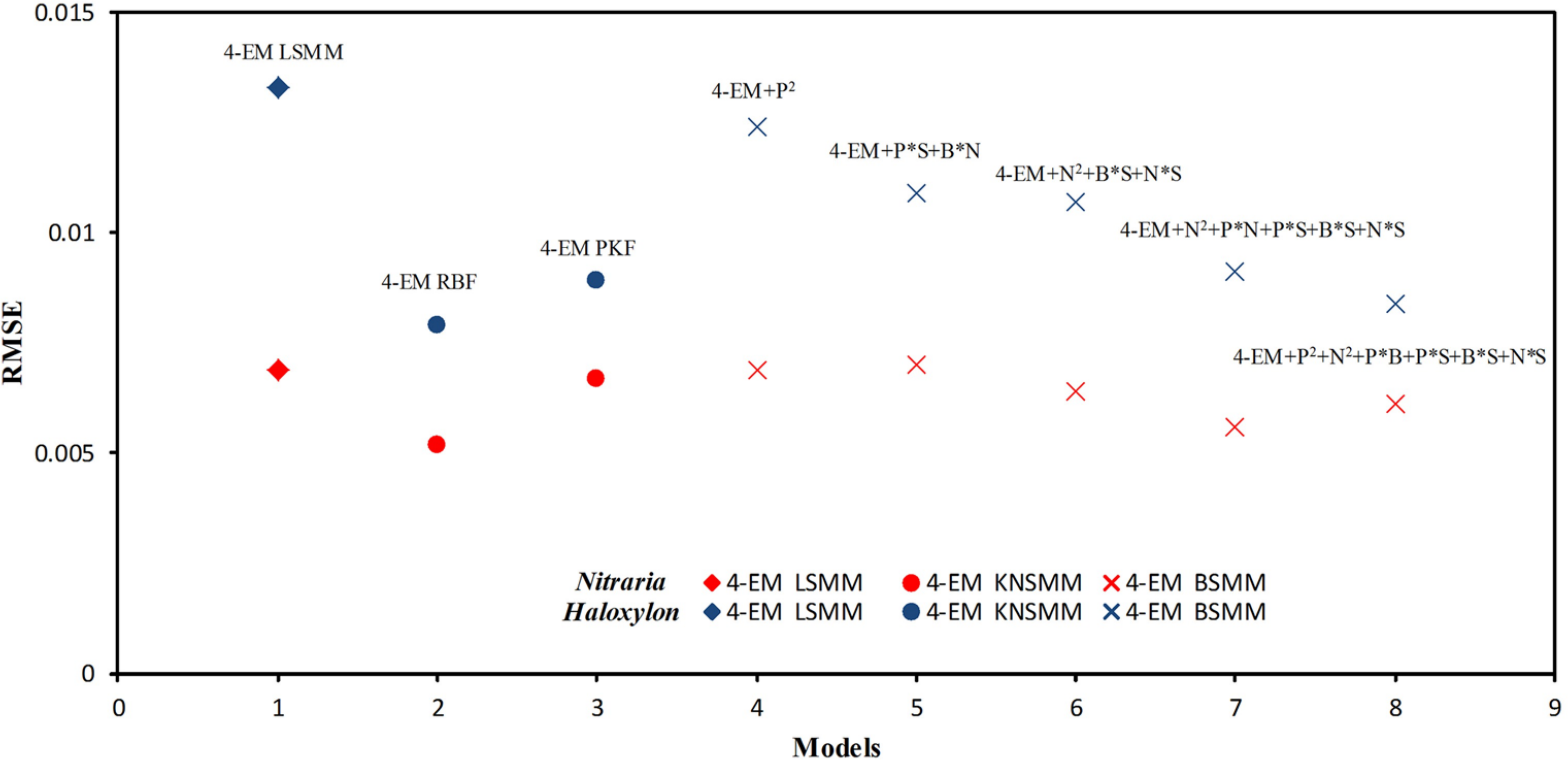
RMSEs for the LSMM, KNSMM, and BSMM, each accounting for different scenario multiple photon scattering effects based on 4-EM.

According to the pixel spectral unmixing results from the *Nitraria* shrub plots and *Haloxylon* plots (Fig 9), the KNSMM was the most appropriate to use in *Nitraria* and *Haloxylon* plots, but the BSMM also yielded reasonable results and is simpler to use. A trade-off between model fit (RMSE) and model simplicity (number of model terms) should ultimately be used to select the most appropriate model. According to Fig 9, based on reliable 4-EM spectral data in the plot showing the precision of simulated pixel spectral curves derived with the LSMM, BSMM, and KNSMM, the curves were similar in the *Nitraria* shrub plots, but slight differences were observed in the *Haloxylon* plots whereby the simulated pixel spectral lines of the KNSMM corresponded better to the measured pixel canopy spectral line than the simulated lines of the LSMM and BSMM.

From the estimation R^2^ and RRMSE of the *f*_pv_ and *f*_npv_ in the *Nitraria* shrub and the *Haloxylon* plots shown in Table 1, Figs 6 and 7, the verification results demonstrated that LSMM could more effectively estimate the fractional cover of PV and NPV in the *Nitraria* shrub, but NSMM made a great progress to estimate the fractional cover of PV and NPV in the *Haloxylon* plots. All experimental verified results indicated that there is multiple interactive photon scattering in the canopy spectra in the *Nitraria* shrub plots and the *Haloxylon* plots, but the scattering influence on the fractional cover estimation of PV and NPV was more outstanding in the *Haloxylon* plots than in the *Nitraria* shrub plots. However, in view of the multiple photon scattering terms between three-dimensional (3D) structures (e.g., PV or NPV) and two-dimensional (2D) structures (e.g., BS or shadows) on the ground surface, the precision of models indicates that multiple photon scattering on the spectral mixture was determined by the canopy structure of tree. Above all, LSMM is the most appropriate model to estimate *f*_pv_ and *f*_npv_ in *Nitraria* shrubs plots, but KNSMM is the best fitting model to estimate the vegetation cover in *Haloxylon* plots.

## Discussion and conclusions

### Discussion

There are advantages and disadvantages to the different spectral unmixing models, LSMMs, BSMMs, and KNSMMs. KNSMM was proved performing best, and BSMM, considering the mutual endmembers scattering explicitly, worked better than the LSMM. Consistent with the previous studies[28, 65, 66], the BSMM could yield clear physical interpretations and understand the spectral mixing relationship than LSMM much better. But BSMM also suffered from a variety of problems, such as over-fitting, collinearity of virtual endmembers[67], especially, the appropriate construction of the virtual multiple photon scattering terms. KNSMMs took an alternative approach through introducing the reproducible kernels in its formulation, and performed spectral unmixing in a high-dimensional feature space so that nonlinear spectral mixture effects could be resolved. From the view of unmixing and fraction estimation accuracy, KNSMM improved obviously compared to BSMM, especially for *Haloxylon* vegetation[43–45, 54]. However, more cautions should be given to the application of KNSMMs, since the appropriate selection of kernel functions and its parameters would play a great influence over the results[47, 60]. In spite of the NSMMs’ relative better performance, LSMMs still could be applied when the non-linear spectral mixing effects was not obviously, with its incomparable advantages of simple computation and definite physical meaning results. Choosing LSMM or NSMM depend on the tradeoff between computational complexity and the specific accuracy requirements.

In the previous studies, the contribution of shadows to the mixed spectra was considered to be only 0–1% or negligible[16, 28, 29, 68]. When shadows were uniformly dark components, while, other scholars [13, 69, 70] had proposed that shadows were important components and could not be neglected in mixed pixel. Thus, there was no consensus on whether shadows should be included or not in unmixing the mixed spectra [71, 72]. In this study, the accuracy of unmixing and the fractional cover estimation would decreased obviously if the shadow endmember was not considered, which proved that the shadow endmember could not be neglected for unmixing the desert vegetation spectra at the plot scale. It was worth noting the variations of the shadow endmembers were not taken into account in this study. However, the darkness differences between shadowed leaves and shaded soil in different time and place, had been found affecting the mixed spectra seriously [69, 70], which should be addressed in the next step.

Multiple photon scattering process was comparatively more likely to happen in the *Haloxylon* plots than in the *Nitraria* shrub plots. According to the BSMM results, the spectral multiple photon scattering processes mainly occurred between PV and NPV, which means that the canopy structure is one of the primarily factors for the photon non-linear scattering. For the two selected desert vegetation type, *Nitraria* shrubs presents planophile canopy, characterized by short height, sparse leaf, which tends to decrease the chance of multiple photon scattering in the canopy. Instead, *Haloxylon* shows erectophile plants canopy characterized by higher height, dense and needle leaf, which is more prone to multiple photon scattering. These conclusions are consistent with the previous studies[73, 74]. Therefore, we can draw the same conclusion as Somers et al [17] that the different canopy structure of the *Haloxylon* and *Nitraria* shrub determines the strength of the nonlinear spectral mixing effects.

### Conclusions

In this study, the nonlinear spectral mixture effects between PV/NPV and bare soil in *Nitraria* shrubs and *Haloxylon*, were investigated through comparing the PV/NPV estimation performance with different LSMMs, NSMMs, based on field measured spectra at the plot scale. Overall, the major conclusions can be summarized as follows.

1. Shadows should not be neglected for modeling the mixed spectra of *Nitraria* shrubs and *Haloxylon*. The 4-EM models, including the shadow endmember, could effectively improve the model unmixing accuracy. The unmixing RMSE (%) were improved by 25.64% and 16.97% in the *Nitraria* shrubs plots and the *Haloxylon* plots respectively. Therefore, the correctness of the selection of the endmembers plays a significant role in improving the unmixing accuracy.
2. Generally, NSMMs work better than LSMM for *f*_pv_ and *f*_npv_ estimations, which means that the non-linear mixing effects do exist in desert vegetation. However, the improvements in *Nitraria* shrubs are not obvious as in *Haloxylon*. For the performance of NSMMs in *Haloxylon* plots, KNSMMs are obviously better than BSMMs. Considering computational complexity and accuracy requirements, the LSMM may be adopted to *Nitraria* shrubs plots for estimating *f*_pv_ and *f*_npv_, but, for *Haloxylon* plots, NSMMs should be used to deal with the obvious non-linear mixing effects.
3. The non-linear mixing effects are closely related to the plant canopy structure, whose strength is greater in vegetation with erectophile canopy (Haloxylon) than with planophile canopy (Nitraria shrubs).
